# Whom Should Be Saved? A Proposed Ethical Framework for Allocating Scarce Medical Resources to COVID-19 Patients Using Fuzzy Logic

**DOI:** 10.3389/fmed.2021.600415

**Published:** 2021-03-22

**Authors:** Heba Saadeh, Maha Saadeh, Wesam Almobaideen

**Affiliations:** ^1^Computer Science Department, King Abdullah II School of Information Technology, The University of Jordan, Amman, Jordan; ^2^Computer Engineering and Informatics, School of Science and Technology, Middlesex University Dubai, Dubai, United Arab Emirates; ^3^Electrical Engineering and Computing Sciences, Rochester Institute of Technology in Dubai, Dubai, United Arab Emirates

**Keywords:** COVID-19, ethical framework, ethical principles, fuzzy logic, resource allocation, scarce resources, WHO ethical guidelines

## Abstract

COVID-19 is a global pandemic that affected the everyday life activities of billions around the world. It is an unprecedented crisis that the modern world had never experienced before. It mainly affected the economic state and the health care system. The rapid and increasing number of infected patients overwhelmed the healthcare infrastructure, which causes high demand and, thus, shortage in the required staff members and medical resources. This shortage necessitates practical and ethical suggestions to guide clinicians and medical centers when allocating and reallocating scarce resources for and between COVID-19 patients. Many studies proposed a set of ethical principles that should be applied and implemented to address this problem. In this study, five different ethical principles based on the most commonly recommended principles and aligned with WHO guidelines and state-of-the-art practices proposed in the literature were identified, and recommendations for their applications were discussed. Furthermore, a recent study highlighted physicians' propensity to apply a combination of more than one ethical principle while prioritizing the medical resource allocation. Based on that, an ethical framework that is based on Fuzzy inference systems was proposed. The proposed framework's input is the identified ethical principles, and the output is a weighted value (per patient). This value can be used as a rank or a priority factor given to the patients based on their condition and other relevant information, like the severity of their disease status. The main idea of implementing fuzzy logic in the framework is to combine more than one principle when calculating the weighted value, hence mimicking what some physicians apply in practice. Moreover, the framework's rules are aligned with the identified ethical principles. This framework can help clinicians and guide them while making critical decisions to allocate/reallocate the limited medical resources during the current COVID-19 crisis and future similar pandemics.

## Introduction

The novel coronavirus 2019 (COVID-19) is a global respiratory pandemic that is highly contagious emerged in Wuhan city of China (1). It had negatively affected all the countries worldwide and disturbed the everyday lives of billions of citizens worldwide. It changed the way people used to work, learn, interact, communicate, and travel (2, 3). The social, economic, and psychological impacts of COVID-19 are essential; however, the physiological impact on the health of the infected individuals and the patients of chronic diseases and/or patients who need emergent care is crucial since it has a direct, apparent, and immediate effect on the health care system of the country (4–8). The COVID-19 fatality rate is alarming (around 107 million causalities and more than two millions death cases, as of 7th of February, 2021), and the death cases had been recorded for all ages and various health conditions (9, 10), especially with the emerging of new strains of COVID-19 (11).

Italy has been one of the first countries severely affected by the spread of COVID-19 outside China. Ten percent of positively tested patients in Italy needed intensive care to overcome the acute respiratory distress syndrome. Moreover, due to the exponential rise of the number of COVID-19 patients, there was an actual risk of running out of Intensive Care Unit (ICU) beds, ventilators, and other medical resources, including face masks and shields (12, 13). Many medical centers in Italy respond to the largest outbreak of COVID-19 outside Asia by developing and applying their response plans (14). As the number of COVID-19 cases is still increasing worldwide, mainly due to the second wave and virus mutations, in addition to the limitation of medical resources, doctors and medical professionals are left with the hardest decision to make: whom should be saved first? The decision to select one patient over the others is affected by principles, which may vary based on cultural, religious, and humanity-related reasons (15–17).

It is vital to have a comprehensive, ethical, and applicable framework/plan to be used when needed for a sudden and massive crisis. Previous pandemics such as influenza also required high demand from the health systems, and protocols to prevent, control, and mitigate the effects of influenza had been provided (18). Similarly, medical centers and hospitals provided recommendations for containing and managing COVID-19, with a particular interest in the best practice of scarce medical resource allocation. The guiding principles discussed by Emanuel et al. (19) to allocate resources during COVID-19 recommended that the ICU workers act in a way that strives to save the most number of lives and/or maximize the life-years saved. This means “allocating scarce resources to patients who are sick enough to benefit but also have the best chance of survival” (20). Although this guidance is essential, it might cause a bias since this principle does not uphold all persons' protected rights (21). According to the World Health Organization (WHO), excluding population groups from being allocated medical resources would be inappropriate (22). Moreover, resource allocation should be guided by well-established and broadly-applicable ethical principles when there is insufficient supply to meet everyone's needs.

DeJong et al. (23) provide practical ethical suggestions to guide clinicians and medical centers while allocating limited medications for COVID-19 inpatients in the US. They suggested four ethical principles: firstly, the benefit from reducing mortality should be assessed using the best available evidence. Allowing policies should be revised as evidence develops, and medications should be prioritized during the shortage. Secondly, the choices of each patient should be respected. However, when there is an insufficient supply of medications, it may not be possible to follow individual patients and their physicians' preferences. Thirdly, in order to avoid discrimination and mitigate health disparities, scarce medications should be allocated fairly. Lastly, allocation policies should be made accountable, responsive to the concerns of those affected, transparent, and comparable to the situation, including the progression of the epidemic and the proportion of the supply and demand of medications. Moreover, Brown and Goodwin (24) pointed out that resource allocation guidance should be in alliance with anti-discriminatory criteria such as disability, socioeconomic status, race, and insurance status.

Favoring young patients (youngest first) was the outcome of an online survey completed by 586 US participants. The aim was to elicit the general public preferences to allocate ventilators for COVID-19 patients (25). This result is consistent with the proposed guiding ethical principles by Emanuel et al. (19), summarized in treating patients equally, prioritizing the worst-off, and maximizing social and individual benefits. This is also aligned with the Italian physicians' guidelines; give higher priority to the young patients when assigning intensive care supplies (16, 17). Another recent study, conducted in Jordan, collected a total of 754 responses from five different public groups: religion scholars (3.9%), physicians (22.0%), medical students (21.5%), allied health practitioners (16.2%), and lay people (36.3%). The survey was based on nine ethical principles for allocating medical resources: sickest-first, waiting list (order), youngest first, service, random, monetary contribution, survival, instrumental value, and individual behavior (26). Four groups (excluding physicians) favor sickest-first despite the age, while the physicians tend to choose combined criteria when allocating the scarce medical resources (26).

Usually, allocating medical resources is carefully assessed per-case in order to ensure the maximum benefits. However, at the time of crises, health care systems experienced extensive pressure and shortened in medical resources, despite the country's wealth. This is what most of the countries faced during the current pandemic, COVID-19, and hence allocating scarce medical resources was not a straight forward process. Combining more than one ethical principle to determine who should be given medical attention might be a good process to follow. This paper aims to identify the most commonly recommended ethical principles and provide recommendations for their applications. Furthermore, propose an ethical framework using fuzzy logic that guides clinicians' decisions in allocating medical resources to COVID-19 patients. This framework gives weight to patients based on five ethical principles identified according to WHO guidelines and state-of-the-art practices proposed in the literature. However, the proposed framework is solely based on the ethical principles applied when allocating the currently available scarce medical resources to the current patients arriving at a hospital. The differences between rich and developing countries and considering per-hospital resources in addition to the availability of resources at different times in the same hospital were not within the scope of the proposed ethical framework.

## Identified Ethical Principles

Based on current state-of-the-art practices proposed in the literature during the COVID-19 pandemic, five ethical principles that were commonly suggested and recommended are identified. The five principles are: anti-discrimination, prioritize the worst off, social effects, patient's history, and clinical evidence.

### Fairness/Equality/Anti-discrimination

Allocate medical resources randomly among eligible patients. Resource allocation should not exclude patients based on race, age, religion, disability, origin, sexual orientation, gender, perceived quality of life, or any other type of discrimination. According to WHO, this principle must promote specific ethical values such as transparency, inclusiveness, consistency, and accountability (22). Transparency means that the decisions and justifications should be made public. Inclusiveness is relayed to allow decisions affected entities to influence the decision-making process and the decision itself. Consistency is to treat all persons in the same categories in the same way. Finally, accountability means that decision-makers should justify their allocation decisions and be held responsible (22).

### Prioritize the Worst Off

To allocate medical resources to those most at risk or those in greatest medical need. This principle can be applied when it maximizes the expected post-treatment life-years. Thus, favoring younger patients or even sickest patients if it maximizes survival years (19).

### Relational/Social Effects

To consider family responsibilities, such as children or elderly caretakers, and people who contributed or will have a potential contribution to the community, such as physicians, clinicians, and healthcare providers. This principle is being referred to as maximizing social benefits.

### Patient's History

Patients already receiving a medical resource and/or drugs for other severe conditions should continue to receive it.

### Clinical Evidence

Medical resource allocation should be evidence-based. This means allocating the resources to patient groups who have been shown by rigorous randomized clinical trials (RCTs) to benefit the most from the treatment provided.

## Recommendations for the Applications of the Identified Ethical Principles

According to the WHO guidelines and the literature's best practices, the main recommendations on how to implement and apply the identified ethical principle are summarized below.

### Fairness/Equality/Anti-discrimination

World Health Organization recommends that “each person's interest should count equally unless there are good reasons that justify the differential prioritization of resources” (22). WHO also advises that fairness must promote specific ethical values such as transparency, inclusiveness, consistency, and accountability (22). The equality principle justifies the allocation of resources by a lottery (22). According to DeJong et al. (23), a “first-come, first-served” approach should be avoided because it disadvantages those who experience barriers to seeking health care. Instead, a random allocation, such as a lottery, is the fairest way for drug allocation among eligible patients.

Moreover, they recommended that scarce medications be allocated fairly and be made accountable, transparent, proportionate to the situation, and responsive to those affected (23). However, from George Washington University Milken Institute, Adnan Hyder has pointed out that random allocation is challenging for patients with a similar prognosis since it assumes agreement among clinicians of prognostic indicators (27). Brown and Goodwin advised that ethical recommendations must be supplemented with explicit guidance against discrimination or an attempt to balance the concern for maximizing prognosis with concerns about social justice (24). Similarly, according to Liddell et al. (21), “principles must uphold the protected rights of all persons.” Unless the patient/legal representative consents or unless ventilation is not clinically indicated, it is considered a criminal offense and a civil wrong to physically remove intubation.

Moreover, this could be a breach of Article 3 of the European Convention on Human Rights, which protects patients from inhuman and degrading treatment (21). DeJong et al. (23) recommended that “prioritization should not exclude patients based on age, disability, religion, race, or ethnicity, national origin, gender, sexual orientation, or perceived quality of life”. However, Scheidegger et al. had recommended that age is a risk factor for mortality and must be taken into account (28). Kirkpatrick et al. (20) had also recommended that it is necessary to have special consideration to ensure fair distributions of medical resources, especially to patients with disabilities. The “first-come, first-served” approach is not the right approach for resource allocation. As stated by Berlinger et al. (29), “a critically ill patient waiting for an ICU bed might be better able to benefit from this resource than a patient already in the ICU whose condition is not improving.” The random ethical principle (26) is highly recommended to ensure fairness. However, the order of registration (first-come, first-serve), monetary (contribution to the costs of the treatment), and youngest first ethical principles should be avoided since these contradict the fairness principle.

### Prioritize the Worst Off

According to the World Health Organization, this principle is appropriate to guide the allocation of resources for people at risk, such as providing vaccines for healthcare providers (22). Emanuel et al. (19) recommended that medical resources “should go first to front-line healthcare workers and those who care for ill patients and those who keep critical infrastructure operating.” Likewise, Scheidegger et al. (28) had recommended that “professionals whose health is at greater risk in the event of infection with the coronavirus are to be especially protected.” Based on their survey, Yousef et al. (26) have highlighted that the sickest patients are recommended to be considered first for scarce medical resources allocation. Moreover, the likelihood to survive the longest is also considered a priority for scarce medical resource allocation.

### Relational/Social Effects

World Health Organization recommends giving priority to those who contributed or will have a potential contribution to the community, such as clinicians, healthcare providers, and first responders (22). Ethical analysis needs to account for relational effects representing a different value for decisions (27). However, a person's relationship with dependents is hard to assess in a crisis, and the assessment risks becoming a judgment of social worth (30). Emmanuel et al. (19) recommend that medical resources “should go first to front-line healthcare workers and others who care for ill patients and who keep critical infrastructure operating.” Similarly, Yousef et al. (26) had considered giving priority to those who have essential roles for keeping society operational or have contributed in the past to the common good.

### Patient's History

For existing FDA-approved medications, DeJong et al. (23) recommended that, with good evidence, patients already receiving the drug for other severe conditions or severe chronic diseases should continue to receive it.

### Clinical Evidence

DeJong et al. (23) recommended that if there is no evidence that patients who suffer from special health conditions such as coronary artery disease, diabetes, and hypertension show a lower level of therapy response compared to other patients, then the former type of patients should be provided with new therapies. Moreover, they suggested that patient groups receive priority if sound evidence emerged that they have more considerable clinical benefits than others (23). This principle can guide the allocation of scarce resources that confer substantially different benefits to different individuals (22). Kirkpatrick et al. (20) had stated that reallocation of medical resources might occur after time-limited trials to see the evidence of recovery or improvement; otherwise, these resources may be reallocated to other patients. Scheidegger et al. (28) had recommended that the highest priority and intensive care should be given to patients whose condition will be improved with it but will suffer without it.

## Proposed Fuzzy Ethical Framework

Allocating scarce medical resources at the time of crises, like COVID-19, is not a straight forward process and holds a bit of uncertainty. It is sometimes hard to apply only one principle, like youngest first, since physicians tend to assess the need by applying more than one principle at once to maximize the benefits. Therefore, a combined criterion (based on more than one principle) is often more preferred. This is highlighted in a recent comprehensive study conducted in Jordan to assess general public opinions regarding allocating scarce medical resources, and physicians tend to choose combined criteria while deciding who should be given medical attention first (26). Based on that, an ethical framework that combined multiple ethical principles to prioritize medical resource allocation decisions is proposed. Fuzzy Logic (31) was used to model this framework to handle the companion uncertainty.

**Figure 1:**
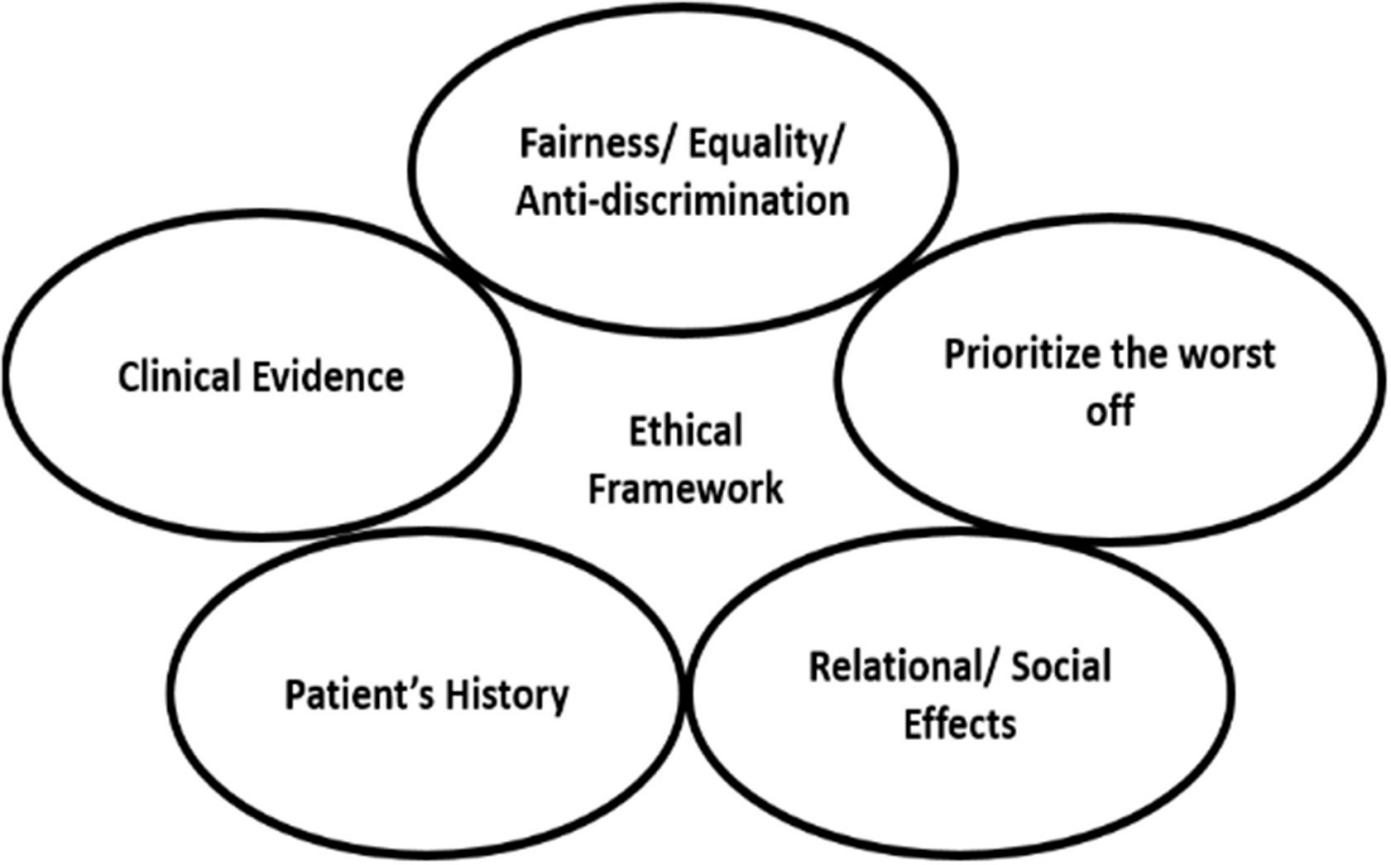
The identified five ethical principles for the proposed fuzzy ethical framework of scarce medical resource allocation.

The proposed fuzzy framework (Figure 1) is centered on the five identified ethical principles (previous section). The idea is to give the patient a weight that can serve as a decision-making factor when scarce medical resources are allocated. This weight is calculated based on the combination of these five ethical principles. For example, if a patient satisfies multiple principles, he/she will get a higher weight than others and hence more likely to get medical resources than others. The fuzzy framework can prioritize the different ethical principles in different settings, and this can vary based on different cultural, religious, and humanity-related factors.

### Fuzzy Logic Overview

The fuzzy logic, first proposed by Zadeh in 1965, is defined as a set of elements with a degree of membership. Thus, instead of having a step value such as 0 or 1, a value can be between 0 and 1, like 0.4. The advantage of fuzzy logic is that it describes the problem in terms of linguistic variables, like age: old or young, making it a powerful tool for managing the vagueness and uncertainty efficiently (31). A Fuzzy inference system is an inference system based on fuzzy logic to infer values using a predefined set of rules. The inference system consists of three main steps: fuzzification, rules evaluation, and defuzzification (32). The inputs and outputs are variables that can have real numbers values. In the fuzzification step, real number inputs are mapped to the fuzzy domain by converting each value into a fuzzy value (linguistic term). For example, if the variable can have any value in the range [0, 1], say 0.3, then the fuzzy value that corresponds to this value is Low. On the other hand, if the input is 0.8, then the fuzzy value is High. Any value in between can be Moderate. In the second step, a set of predefined rules are evaluated. The rule has the following format assuming ***n***fuzzy values for the input variable and ***m***fuzzy values for the output variable:

$$\begin{array}{l} \begin{array}{c} IF\left\{Input-variable\;is\;Value_{n}\right\}THEN\\ \left\{Output-Variable\;is\;Value_{m}\right\} \end{array} \end{array}$$

The previous step's fuzzy output value is mapped back to the real numbers' domain in the last step.

### Details of the Proposed Fuzzy Framework

**Figure 2:**
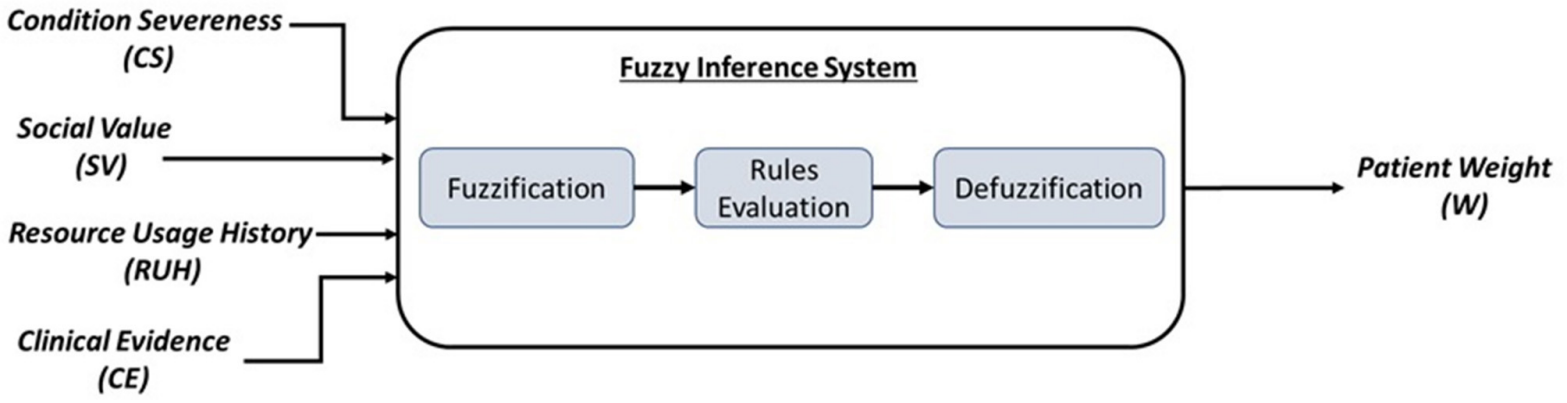
The proposed fuzzy ethical framework components. Inputs: four ethical principles. Processing: three main stages. Output: a weight or decision factor per patient to determine his/her priority in receiving a medical resource.

The proposed ethical framework that is based on the fuzzy logic (Figure 2) takes the following inputs:

1. Condition Severeness (CS): this input is related to the “prioritize the worst off” ethical principle. This input's value is determined by the physicians/clinicians based on the patient's disease condition. The value is between 0 and 1. For example, values 0.3 or less indicate a non-severe condition, while 0.8 or more indicate a severe condition and any values in between indicate moderate condition.
2. Social Value (SV): this input is related to the “relational/social effects” ethical principle. This input's value is determined by the physicians/clinicians based on the patient's social impact. The value is between 0 and 1. For example, a nurse who is the only breadwinner for his/her family can be assigned a social value of 0.8, which indicates a high social impact, i.e., healthcare provider and family support, on the other hand, the social value for a single nurse can be 0.5 indicates a lower social impact, i.e., healthcare provider only.
3. Resource Usage History (RUH): this input is related to the “patient's history” ethical principle. This input's value is determined by the physicians/clinicians based on the patient's record on receiving a particular medical resource. The value is between 0 and 1. For example, a patient who is already receiving a medical resource can be assigned a RUH value of 0.8, which indicates a high priority to continue receiving the medical resource. On the other hand, a value of 0.3 indicates that the patient received medical resources in the past. A value of 0 indicates no previous record of receiving a particular medical resource.
4. Clinical Evidence (CE): this input is related to the “clinical evidence” ethical principle. The value of this input is determined by the physicians/clinicians based on any evidence of the benefit of receiving a particular medical resource. The value is between 0 and 1. For example, a patient who showed recovery or improvement evidence can be assigned a value of 0.8, which indicates a high priority to receive the medical resource. On the other hand, a value of 0.3 indicates that the patient's condition is improving slowly, thus, a lower priority to receive it. A value of 0 indicates no improvement of patient condition is noticed after receiving the medical resource.

The inference system's output is a weighted value (W), which indicates the level of the combined interaction between the four different ethical principles that were met. This output is calculated as a result of predefined rules (shown below). This weighted value can take five fuzzy values: VeryLow (VL), Low (L), Moderate (M), High (H), and VeryHigh (VH). These fuzzy values are then mapped to a value between 0 and 1. Below is an example of some fuzzy rules proposed for the ethical framework.

Fuzzy Inference Rules

*IF* {*CS is H* & *SV is H* & *RUH is H* & *CE is H*} *THEN* {*W is VH*}

*IF* {*CS is H* & *SV is H* & *RUH is H* & *CE is L*} *THEN {W is H}*

*IF* {*CS is H* & *SV is H* & *RUH is L* & *CE is L*} *THEN {W is M}*

*IF* {*CS is H* & *SV is L* & *RUH is L* & *CE is L*} *THEN {W is L}*

*IF* {*CS is L* & *SV is L* & *RUH is L* & *CE is L*} *THEN {W is VL}*

- According to the first rule, if all the four ethical principles were favorably satisfied, then the weight is VeryHigh.
- According to the second rule, if three ethical principles were favorably satisfied, then the weight is High
- According to the third rule, if two ethical principles were favorably satisfied, then the weight is Moderate
- According to the fourth rule, if only one ethical principle was favorably satisfied, then the weight is Low
- And according to the last rule, if all the four ethical principles were not satisfied, then the weight is VeryLow

Note that the “Fairness/Equality/Anti-discrimination” ethical principle is implicitly satisfied by not considering the patient's age and gender in the framework. Moreover, this ethical principle is also satisfied when two or more patients have equal weights; accordingly, a random selection can be applied.

## Conclusion

The massive disruption caused by the COVID-19 pandemic had uncovered the lack of readiness in the health systems regarding staff members and medical resources. The rapid and increasing number of infected patients in a short time and the severe medical complications accompanying the disease overwhelmed the health care infrastructure of many counties. Thus, clinicians had been in desperate need of practical and ethical recommendations to guide them while allocating and reallocating scarce resources for and between COVID-19 patients. This research identified the most commonly recommended ethical principles in accordance with WHO guidelines and literature's best practices and provide recommendations for their applications. Furthermore, it proposed an ethical framework based on fuzzy logic that can help clinicians and guide them in their decisions while allocating limited medical resources, like ICU beds and ventilators, to COVID-19 patients. This framework is aligned with the identified ethical principles and can also be applied in a similar future pandemic. Finally, expanding the current proposed fuzzy framework to consider not only ethical principles but also other per-hospital resource availability and other different restrictions globally worth investigating.

## Data Availability Statement

The original contributions presented in the study are included in the article/Supplementary Material, further inquiries can be directed to the corresponding author/s.

## Author Contributions

All authors listed have made a substantial, direct and intellectual contribution to the work, and approved it for publication.

## Conflict of Interest

The authors declare that the research was conducted in the absence of any commercial or financial relationships that could be construed as a potential conflict of interest.

## Supplementary Material

Supplementary Figure 1 The proposed ethical framework using the Mamdani inference system implemented in Matlab.

Supplementary Figure 2 The membership functions of input variables.

Supplementary Figure 3 The membership functions of the output variable.

Supplementary Figure 4 Sample of the fuzzy inference rules.

Supplementary Figure 5 The output weight variable is VeryHigh when all ethical principles are satisfied for a particular patient.

Supplementary Figure 6 The output weight variable is Low when a single ethical principle is satisfied for a particular patient.

## Appendix

This appendix discusses the implementation of the proposed ethical framework using the Matlab Fuzzy Logic Toolkit. Firstly, we need to implement the structure of the fuzzy inference system. The proposed ethical framework consists of four input variables that correspond to the identified ethical principles, which are Condition Severeness (CS), Social Value (SV), Resource Usage History (RUH), and Clinical Evidence (CE) and one output variable, which is the Weight (W). Supplementary Figure 1 shows the proposed framework as implemented by Matlab using Mamdani fuzzy inference. Each input variable is mapped to the following fuzzy values:

- Low is represented by a trapezoidal membership function, which takes four values to specify the range (0, 0, 0.1, 0.4). This means that any input value in this range will be considered as Low.
- Moderate is represented by a triangular membership function, which takes three values to specify the range (0.25, 0.5, 0.75). This means that any input value in this range will be considered as Moderate.
- High is represented by a trapezoidal membership function, which takes four values to specify the range (0.6, 0.9, 1, 1). This means that any input value in this range will be considered as High.

As illustrated in Supplementary Figure 2, the membership functions are overlapped to allow inputs to take two values in different membership functions. For example, an input value of 0.3 is considered both Low and Moderate since it intersects with both functions. The output variable W is represented in five fuzzy values (Supplementary Figure 3) as the following:

- VeryLow is represented by a trapezoidal membership function, which takes four values to specify the range (0, 0, 0.15, 0.3). This means that any output value in this range will be considered as VeryLow.
- Low is represented by a triangular membership function, which takes three values to specify the range (0.15, 0.3, 0.45). This means that any output value in this range will be considered as Low.
- Moderate is represented by a triangular membership function, which takes three values to specify the range (0.35, 0.5, 0.65). This means that any output value in this range will be considered as Moderate.
- High is represented by a triangular membership function, which takes three values to specify the range (0.55, 0.7, 0.85). This means that any output value in this range will be considered as High.
- VeryHigh is represented by a trapezoidal membership function, which takes four values to specify the range (0.7, 0.85, 1, 1). This means that any output value in this range will be considered as VeryHigh.

Secondly, after implementing the fuzzy inference inputs and output, we added the fuzzy rules. Supplementary Figure 4 shows a sample of these rules. The fuzzy values for input variables will be matched with each rule. The rule will be applied when the condition part is satisfied (fully matched with the fuzzy inputs values); otherwise, the rule will not be selected. Supplementary Figure 5 shows the applied rule when inputs have high values (when all ethical principles are met), which is rule number 1. As illustrated, the weight, in this case, is VeryHigh = 0.865. The scenario in Supplementary Figure 6 is applied when the patient satisfies only a single ethical principle in which the weight is Low = 0.3.
