# Supplementary figures and images for: Whom Should Be Saved? A Proposed Ethical Framework for Allocating Scarce Medical Resources to COVID-19 Patients Using Fuzzy Logic
Source: Front Med (Lausanne). 2021 Mar 22;8:600415. doi: 10.3389/fmed.2021.600415 (PMC8020032; doi:10.3389/fmed.2021.600415)

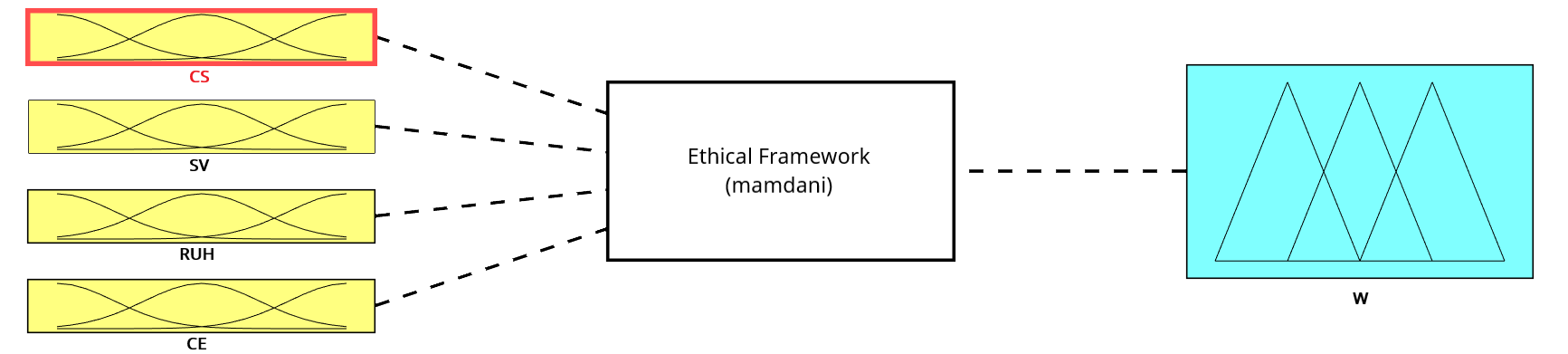

Supplement: Supplementary Figure 1 — The proposed ethical framework using the Mamdani inference system implemented in Matlab. [file Image_1.tiff]

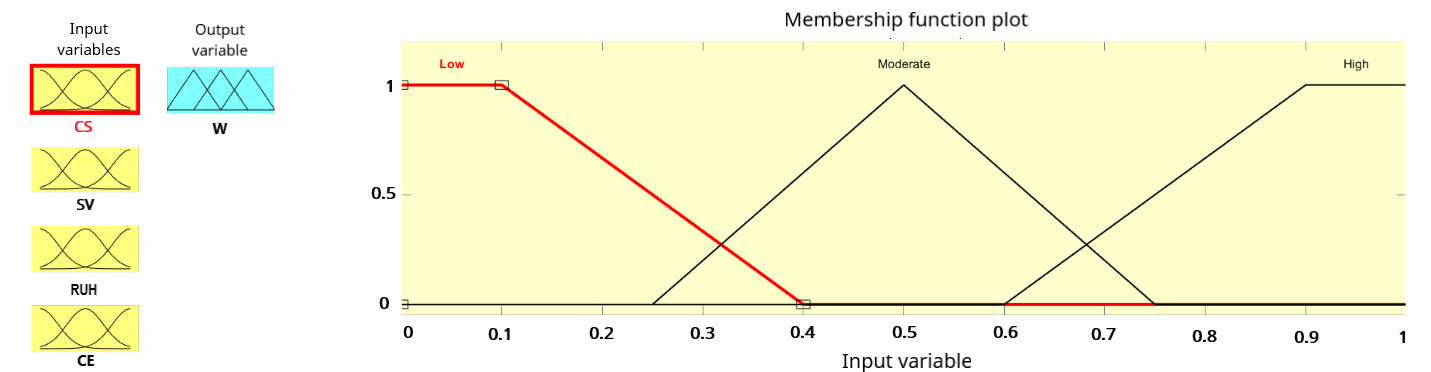

Supplement: Supplementary Figure 2 — The membership functions of input variables. [file Image_2.tiff]

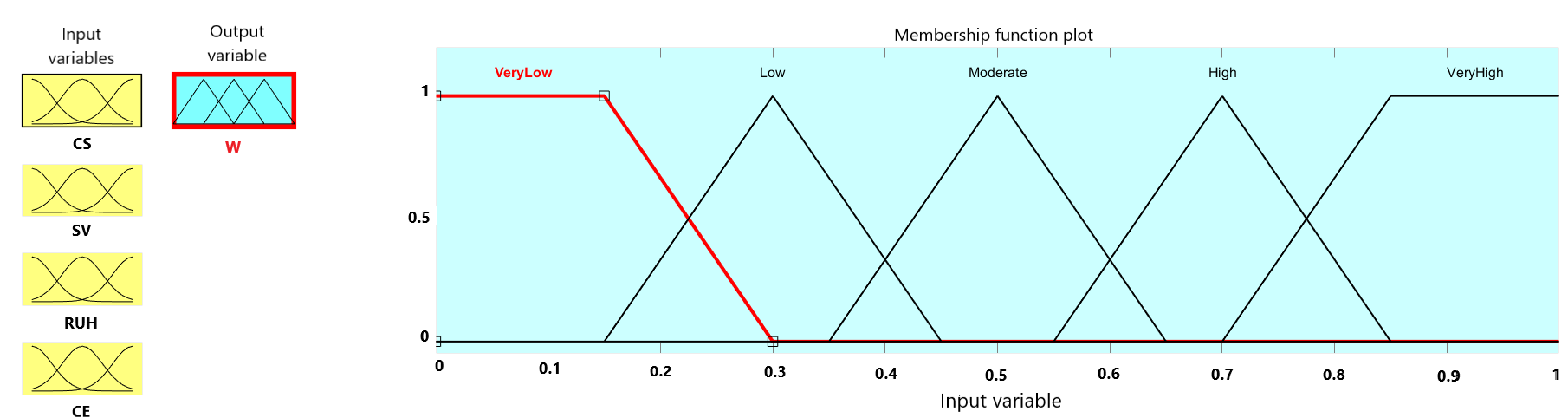

Supplement: Supplementary Figure 3 — The membership functions of the output variable. [file Image_3.tiff]

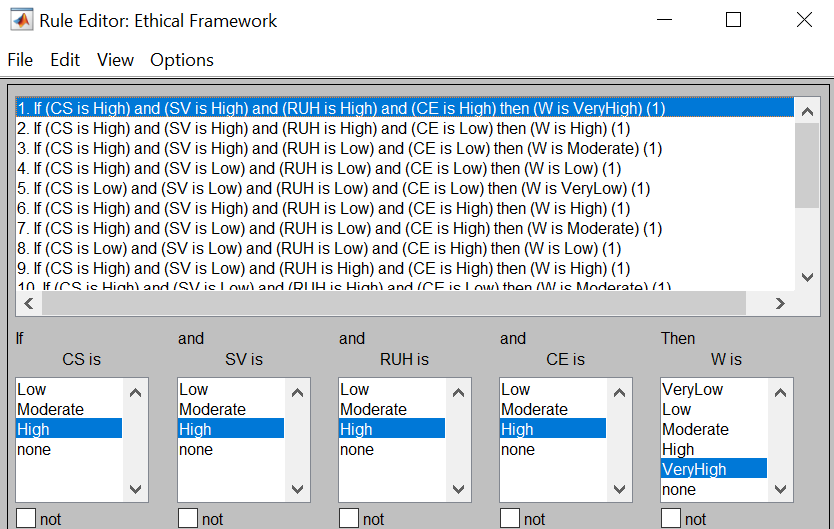

Supplement: Supplementary Figure 4 — Sample of the fuzzy inference rules. [file Image_4.tiff]

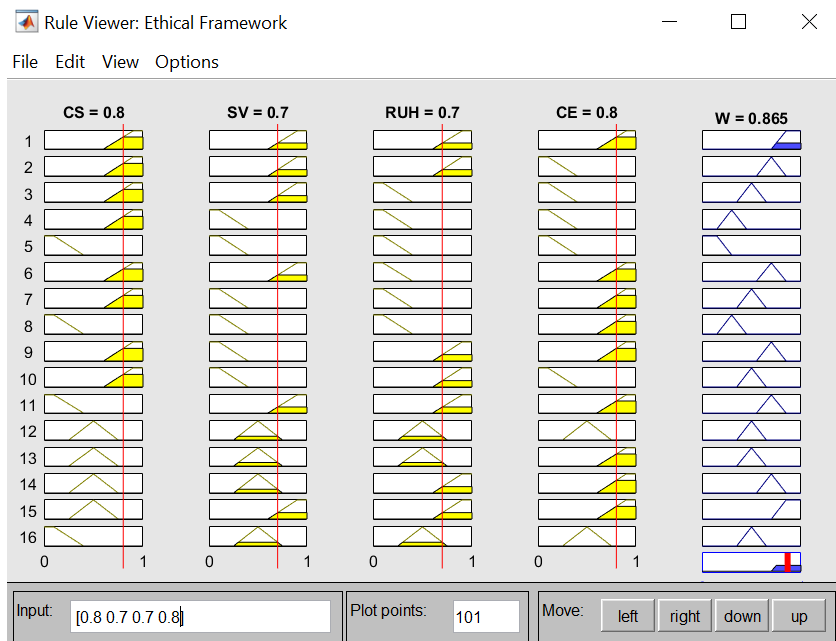

Supplement: Supplementary Figure 5 — The output weight variable is VeryHigh when all ethical principles are satisfied for a particular patient. [file Image_5.tiff]

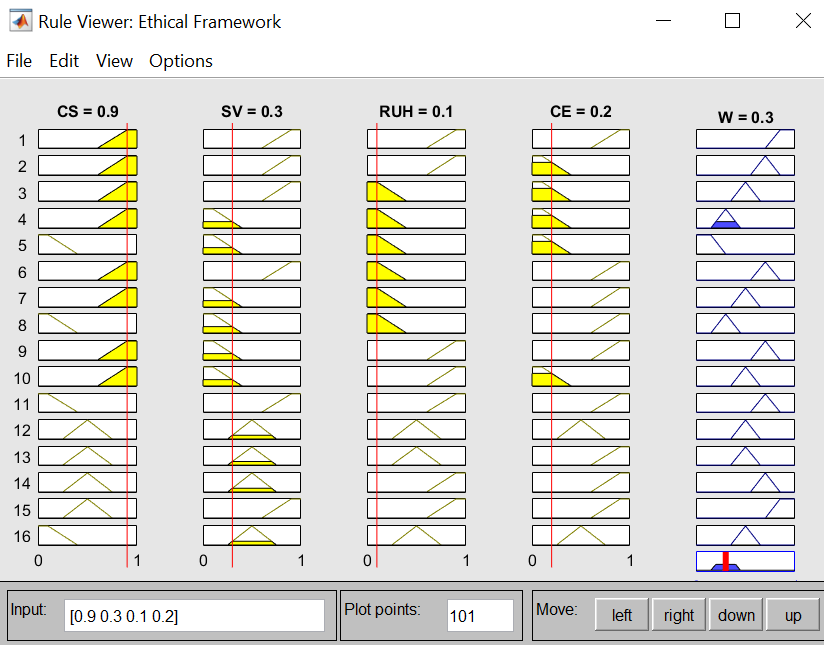

Supplement: Supplementary Figure 6 — The output weight variable is Low when a single ethical principle is satisfied for a particular patient. [file Image_6.tiff]
